# Effects of Different Shading Treatments on the Biomass and Transcriptome Profiles of Tea Leaves (*Camellia sinensis* L.) and the Regulatory Effect on Phytohormone Biosynthesis

**DOI:** 10.3389/fpls.2022.909765

**Published:** 2022-06-24

**Authors:** Zhou-Tao Fang, Jing Jin, Ying Ye, Wei-Zhong He, Zai-Fa Shu, Jing-Na Shao, Zhu-Sheng Fu, Jian-Liang Lu, Jian-Hui Ye

**Affiliations:** ^1^Tea Research Institute, Zhejiang University, Hangzhou, China; ^2^Zhejiang Agricultural Technical Extension Center, Hangzhou, China; ^3^Lishui Institute of Agriculture and Forestry Sciences, Lishui, China; ^4^Zhejiang Minghuang Natural Products Development Co., Ltd., Hangzhou, China

**Keywords:** *Camellia sinensis* (L.) O. Kuntze, light intensity, light spectral composition, biomass, transcriptome, phytohormones

## Abstract

Our previous study showed that colored net shading treatments had comparable effects on the reduction of bitter and astringent compounds such as flavonol glycosides in tea leaves, compared with black net shading treatment, whereas the effects on the biomass and phytohormones are still unclear. In this study, we investigated the phytohormone and transcriptome profiles of tea leaves under different shading treatments, using black, blue, and red nets with the same shade percentages. The bud density, fresh weight of 100 buds, and yield under blue net shading treatments were greatly elevated by 2.00-fold, 1.24-fold, and 2.48-fold, compared with black net shading treatment, while their effects on flavonoid composition were comparable with black net shading treatment. The transcriptome profiles of different shade net-treated samples were well resolved and discriminated from control. The KEGG result indicated that the pathways of phenylpropanoid biosynthesis, MAPK signaling pathways, and plant hormone signal transduction were differentially regulated by different shading treatments. The co-expression analysis showed that the contents of salicylic acid and melatonin were closely correlated with certain light signal perception and signaling genes (*p* < 0.05), and UVR8, PHYE, CRY1, PHYB, PHOT2, and HY5 had more close interactions with phytohormone biosynthetic genes (*p* < 0.05). Our results suggest that different shading treatments can mediate the growth of tea plants, which could be attributed to the regulatory effect on phytohormones levels, providing an instruction for the production of summer/autumn tea and matcha.

## Introduction

Tea is a popular non-alcoholic drink over the world. The ground powder and extract of tea leaves are common raw materials and ingredients of food products, such as bakery, ready-to-drink tea, and matcha latte. Tea is produced from the leaves of the plant *Camellia sinensis* (L.) O. Kuntze, which contain abundant secondary metabolites, such as catechins, flavonol glycosides, and caffeine (Samynathan et al., 2021). Many of the metabolites are bitter and/or astringent, forming the unique flavor of tea (Ye et al., 2022). As raw materials and ingredients of food products, the bitterness and astringency of tea need reduction so as to endow foods with better flavor (Ye et al., 2022). In addition to tea breeding (Xia et al., 2020), agronomic practices are efficient to reduce the bitter and astringent taste of tea, e.g., shading treatment and fertilization (Ye et al., 2022). Black net shading treatment is effective to decrease the accumulation of bitter and astringent compounds like flavonol glycosides in tea leaves (Jin et al., 2021). However, the growth of tea plants is also attenuated or even adversely impacted due to the weak light condition. Long-time shading treatment leads to the greatly reduced biomass of young shoots per unit area or growth rate of young shoots (Sano et al., 2018; Ye et al., 2022). In many tea plantations for producing matcha, heavy fertilization of organic fertilizer is implemented to keep the good growth status of tea plants. The utilization of shade nets, high input of organic fertilizer, and the decreased production yield cause the relatively high production cost of matcha.

Light condition is an important environmental factor in the growth and metabolism of tea plants (Homma et al., 2011). UV-light (below 400 nm), visible light (400–710 nm), and infrared radiation (710–1,000 nm) comprise the sunlight, and the visible light spectrum consisted of blue light (400–495 nm), green light (495–570 nm), yellow light (570–590 nm), and red light (590–710 nm) (Zoratti et al., 2014). Plants have the perception of light, including light intensity, direction, specific wavelengths, and photoperiod. Different types of light receptors were found in plants, including blue-light receptor phototropins (PHOTs), blue light, UV-A receptor cryptochromes (CRYs), UV-B receptor, UV resistance locus 8 (UVR8), and phytochromes (PHYs) that perceive red/far-red light signals (Paik and Huq, 2019). Elongated hypocotyl5 (HY5), suppressor of PHYA (SPA), and constitutively photomorphogeni1 (COP1) are important components of light signal transduction (Yadav et al., 2020). Up to date, the regulatory effects of light intensity and light quality on tea plants mainly focus on the secondary metabolism in tea leaves (Zheng et al., 2019; Lin et al., 2021; Ye et al., 2021). It was reported that blue light elevated the expressions of *CRY2/3, SPAs, HY5*, and *R2R3-MYBs*, leading to the enhanced accumulation of anthocyanins and catechins in tea plants (Zheng et al., 2019). UV promoted the accumulation of flavonol glycosides in tea plants, which was associated with the upregulated expressions of *UVR8* and *HY5* (Lin et al., 2021; Ye et al., 2021). In addition, the biosynthesis and transportation of hormones in plants can also be affected by the light condition (Weller et al., 2009; Ding et al., 2011; Yi et al., 2020). Light regulated the biosynthesis of gibberellin in pea by mediating COP1/HY5 pathway (Weller et al., 2009). Light promoted the biosynthesis of jasmonate in *Arabidopsis* to regulate photomorphogenesis (Yi et al., 2020). The levels of hormones in plants are related to the growth rate, biomass, and resistance of plants. The information about the effect of light condition on the production yield of tea plants is scarce. In our previous study, flavonol glycosides were greatly reduced under both black and blue net shading treatments, while the production yield of tea plants under blue net shading treatment was apparently higher than that of black net shading treatment (Jin et al., 2021). The impact of light condition on the biomass of tea plants and the light-mediated regulatory effect on the biosynthesis of phytohormones in tea plants need further investigations.

In this study, the effects of different shading treatments (black, blue, and red nets with the same shade percentages) on the biomass of tea plants “Longjing 43” were investigated, in terms of bud density, weight of 100 buds, and production yield. In addition to the composition of flavonoids, the biosynthesis of phytohormones in tea leaves under different shading treatments was studied and compared using the combination of phytohormone and transcriptome analyses. Kyoto Encyclopedia of Genes and Genomes (KEGG) pathway enrichment analysis was performed to reveal the biological and metabolic changes of tea leaves under different shading treatments. Co-expression analysis was carried out to explore the correlations of light signal transduction-related gene expressions with the phytohormone levels and the transcriptional levels of phytohormone biosynthetic genes.

## Materials and Methods

### Chemicals and Reagents

Individual catechins (EGCG, EGC, ECG, EC, GCG, GC, CG, C, all > 95%) and flavonol (myricetin, quercetin, kaempferol, all > 95%) were bought from Sigma-Aldrich (Shanghai, China). Acetonitrile and methanol (HPLC grade) were bought from Merck KGaA Company (Darmstadt, Germany). Ethanol was purchased from Sinopharm Chemical Reagent Co., Ltd. (Shanghai, China). The HPLC grade phytohormones including abscisic acid (ABA, ≥98%), gibberellin 1 (GA1, 95%), gibberellin 3 (GA3, 98%), indole-3-acetic acid (IAA, ≥98%), *trans*-zeatin-riboside (tZR, ≥98%), zeatin (Z, 95%), jasmonic acid (JA, 95%), melatonin (≥95%), and salicylic acid (SA, 95%) were all purchased from Shanghai Yuanye Bio-Technology Co., Ltd. (Shanghai, China).

### Shading Treatment and Sampling

*Camellia sinensis* cv. Longjing 43 (4 years old, height 0.78 m), growing in the Songyang Tea Plantation of Lishui Academy of Agricultural Sciences (Lishui County, Zhejiang, China, 28°577′N, 119°377′ E), was used for the study. Shortly after light pruning, the polyethylene nets, including black, blue, and red nets (4 m × 6 m, the shade percentages of 95%), were used to shade tea plants from the middle of June to the end of June 2021, at the height of 2 m above ground level. All the shade nets were bought from Taizhou Huiming Shade Net Co., Ltd. (Taizhou, China). The shading treatments were implemented according to the method of our previous work (Jin et al., 2021; Ye et al., 2021). After shading for 15 days (29 June 2021), the tea plants were at the stage of two leaves and one bud, and the second leaves basipetal from the apical bud were randomly collected. The tea plants with edge effect were avoided. Three independent biological replicates were randomly collected, and 3–5 tea plants were used for each biological repeat. The fresh tea leaves were immediately placed in liquid nitrogen for 30 min. The tea leaves of the same biological replicate were mixed quickly and divided evenly into three portions for transcriptomic, flavonoid, and phytohormone analyses. All the samples were stored at −80°C prior to these analyses.

### Illumination and Light Measurement

The parameters of light condition were measured on the same date of harvest. The light intensity at the height of tea shoots was measured by light intensity sensors of TOP Instruments (Zhejiang Top Instrument Co., Ltd., Hangzhou, China), and the UV intensity was measured by the TENMARS UVAB light meter (TENMARS Electronics Co., Ltd, Taiwan, China). The intensity of the visible spectrum was recorded by HopooColor OHSP-350C Illumination Analyzer (HopooColor Technology Co., Ltd., Hangzhou, China).

### Biomass Measurements

#### Bud Density

The number of buds in a certain area of tea row (length 1 m, width 1.05 m) was counted. The bud density was calculated based on the number of buds per square meter. For each treatment, three different areas (length 1 m, width 1.05 m) in the same tea row were employed for the bud density measurements.

#### Weight of 100 Buds

The young shoots were manually plucked at the plucking standard of two leaves and one bud, and every 100 young shoots under different shading treatments were weighed.

#### Production Yield (g/m^2^)

For each treatment, the young shoots in a certain area of tea row (length 1 m, width 1.05 m) were harvested at the standard of two leaves and one bud and weighed, and the production yield per square meter was calculated. For each treatment, three different areas (length 1 m, width 1.05 m) in the same tea row were employed for the production yield measurements.

### Phytohormone Analysis

The phytohormones were extracted and analyzed by a commercial service company (Gene Denovo Biotechnology, Guangzhou, China) according to the method reported by Ye et al. (2021). After extraction and purification, the phytohormones were analyzed by the UHPLC-MS/MS system consisting of Waters Acquity UPLC and AB SCIEX 5500 QQQ-MS. A multiple reaction monitoring (MRM) mode was used for quantifying ABA, GA3, GA1, IAA, JA, SA, Z, melatonin, and tZR. Data acquisition, peak integration, and calculations were carried out using the MultiQuant software.

### Determination of Flavonoids

Flavonoids were extracted and analyzed according to the method reported by Zheng et al. (2018). Fresh tea leaves were freeze-dried, ground, and sifted through a 0.45-mm sifter. The ground tea sample (0.15 g) was extracted with 25 ml of 50% (v/v) ethanol solution at 100 rpm and 70°C for 30 min. After centrifugation at 12,000 rpm and 4°C for 15 min, the supernatants were analyzed using ultra-high-performance liquid chromatography–diode array detector–tandem mass spectrometry (Waters Corporation, Milford, MA, USA, UPLC–DAD–MS) as reported by Zheng et al. (2018). Catechins were quantified by authentic standards, and flavonol glycosides were quantified by their aglycones.

### Transcriptomic and Bioinformatic Analyses

The RNA isolation and sequencing were conducted by Gene Denovo Biotechnology Co., Ltd. (Guangzhou, China). The total RNA was extracted using the TRIzol reagent kit (Invitrogen, Carlsbad, CA,USA) according to the manufacturer's protocol. The eukaryotic mRNA was enriched by oligo (dT) beads and further fragmented into short fragments. The obtained short fragments were reverse-transcripted into cDNA by using NEBNext Ultra RNA Library Prep Kit for Illumina (NEB #7530,New England Biolabs, Ipswich, MA, USA). After end repaired, A base added, and ligated to Illumina sequencing adapters, the ligated products were selected by agarose gel electrophoresis, PCR amplified, and purified using the AMPure XP Beads (1.0X) to obtain the library. The cDNA library was sequenced using Illumina Novaseq6000. The raw reads were filtered by fastp (version 0.18.0) to obtain the high-quality clean reads by removing adaptor, duplication, and ambiguous sequences (reads with above 10% “N” rate), as well as low quality reads containing more than 50% of low quality (*Q*-value ≤ 20) bases. The reference tea genome of “Longjing 43” (Wang et al., 2020) was used to map clean reads using HISAT2. 2.4, with “-rna-strandness RF” and other parameters set as a default (Pertea et al., 2016; Wei et al., 2018). The mapped reads of each sample were assembled by StringTie v1.3.1. The differentially expressed genes (DEGs) in the RNA-Seq dataset were identified by DESeq2 (| log_2_ fold change | > 1, FDR < 0.05) based on the read counts.

### Quantitative Real-Time Polymerase Chain Reaction Analysis

For quantitative real-time polymerase chain reaction (RT-qPCR) analysis, an RNA sample (1 μg) was converted into first-strand cDNA using PrimeScript™ RT Reagent Kit with gDNA Eraser (TaKaRa Biotechnology Co., Ltd., Dalian, China). The specific primers of selected genes in Supplementary Table 1 were designed by NCBI Primer-BLAST according to the genome sequences of *Camellia sinensis* cv. Shuchazao (Wei et al., 2018). The qPCR cycling was carried out by Applied Biosystems™ StepOnePlus™ Real-Time PCR System (Applied Biosystems™ ABI, Carlsbad, CA, USA) based on the introduction of PowerUp™ SYBR™ Green Master Mix (Thermo Fisher Scientific Inc., Carlsbad, CA, USA): 95°C for 120 s and 40 cycles at 95°C for 3 s, annealing at 60°C for 30 s. The relative expression level of each gene was calculated by 2^−Δ*ΔCt*^ method, using β-actin as an endogenous control. Technical duplicates were employed for each biological replicate.

### Data Analysis

All the tests were repeated three times, and the mean value ± SD was presented. The significant difference analysis was carried out by the SAS System for Windows version 8.1 (SAS Institute Inc., Cary, NC, USA), using a Tukey's test. The Origin 9.1 (Originlab Corporation, Northampton, MA, USA) was used for heatmap plotting and correlation analysis. Principal component analysis (PCA) was conducted using the Minitab 17 statistical software (Minitab. LLC, State College, PA, USA). The bubble diagrams of KEGG were drafted on the online platform of OmicShare tools (http://www.omicshare.com/tools). The co-expression results were visualized by Cytoscape (v3.7.2).

## Results

### Effects of Shading Treatments on the Biomass of Tea Leaves

Different shading treatments altered the light condition underneath. The visible spectrum as well as the intensities of light and UV are shown in Supplementary Figure 1. The effect of shading treatments on the light condition underneath was in an agreement with our previous study (Ye et al., 2021). Table 1 shows the biomass parameters of tea leaves under different shading treatments. Obviously, the tea plants naturally growing in the sun (CK) had the greatest biomass, in terms of bud density (383 buds/m^2^), fresh weight of 100 buds (15.5 g), and production yield (67.2 g/m^2^) at the plucking standard of two leaves and one bud, while the lowest biomass was achieved for black net shading treatment (bud density of 173 buds/m^2^, 100 bud fresh weight of 11.8 g, and production yield of 23.0 g/m^2^). The biomass of blue and red nets-treated tea plants was in between. Thus, shading treatment greatly reduced the bud density, bud weight, and the yield of young shoots. The biomass of blue and red net shading treatments was significantly higher than that of the black net shading treatment, with bud density and production yield being around two-fold and 2.5-fold of those under black net shading treatment. The fresh bud weights were increased by 23.7% and 19.5% under blue and red net shading treatments, compared with the black net shading treatment. Therefore, the biomass of tea plants was closely related to the growth light condition.

**Table 1:** The biomass parameters of tea plants under different shading treatments.

**Treatment^**a**^**	**Bud density (bud/m^**2**^)**	**Fresh weight of 100 buds (g)**	**Yield (g/m^**2**^)**
Control	383 ± 2a	15.5 ± 0.1a	67.2 ± 1.6a
Black net shading treatment	173 ± 2c	11.8 ± 0.1c	23.0 ± 0.6c
Blue net shading treatment	347 ± 9b	14.6 ± 0.5b	57.0 ± 1.4b
Red net shading treatment	344 ± 4b	14.1 ± 0.2b	54.7 ± 1.3b

^a^*The plucking standard was two leaves and one bud. Data were present as mean ± SD (n = 3). Data with different alphabetic letters (a, b, c) in the same column were significantly different at p < 0.05*.

### Effects of Shading Treatments on the Levels of Phytohormones in Tea Leaves

**Figure 1:**
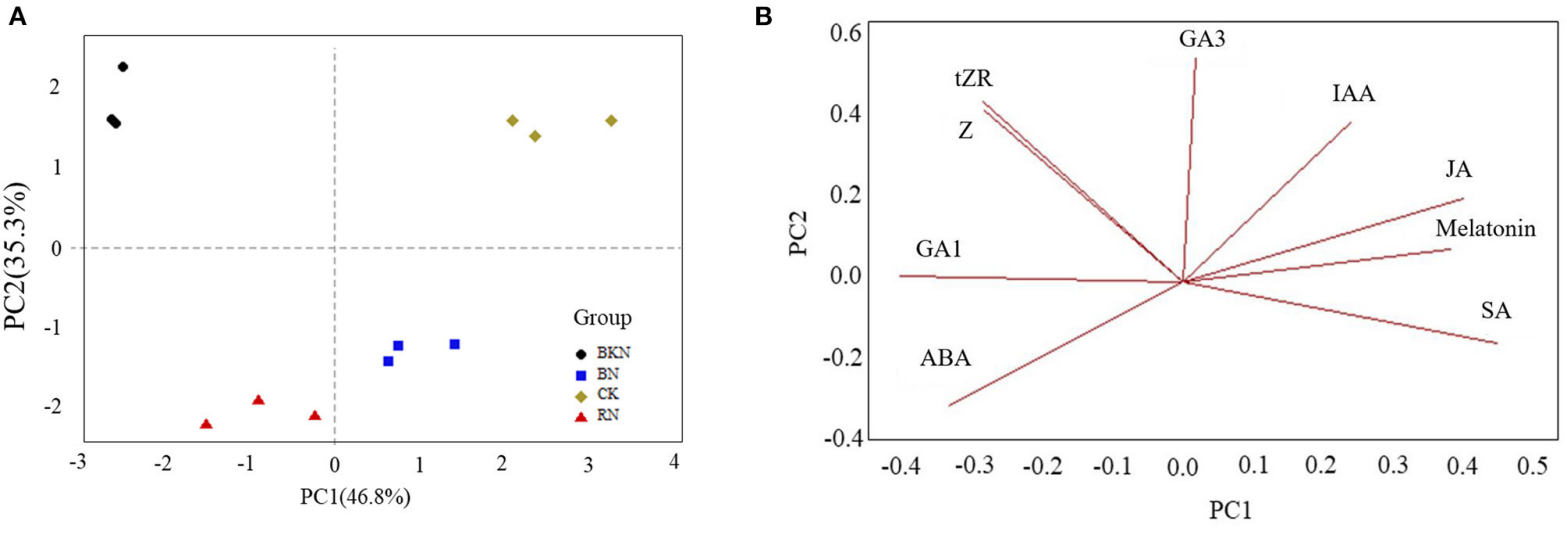
The PCA score plot **(A)** and loading plot **(B)** of tea leaves under different shading treatments based on the levels of endogenous phytohormones.

The growth of tea plants is affected by phytohormone levels, leading to different biomass. Supplementary Table 2 shows the levels of endogenous phytohormones in tea leaves under different shading treatments, including ABA, GA1, GA3, IAA, JA, melatonin, SA, tZR, and Z. Figure 1 shows the PCA score plot and loading plot of tea leaves under different shade treatments based on the levels of endogenous phytohormones. The first two principal components (PC) accounted for 82.1% of the total variance (PC1 = 46.8%, PC2 = 35.3%). Different shading-treated samples were clustered and well discriminated from other samples. Specifically, CK and BN were located in the positive direction of PC1, while BKN and RN were located in the negative direction of PC1 (Figure 1A). Besides, CK and BKN were distributed in the positive direction of PC2, while BN and RN were distributed in the negative part of PC2 (Figure 1A). Thus, PC2 reflected the impact of light spectral composition. The loading plot showed that CK was discriminated from the shading-treated samples due to its highest levels of IAA and JA, while BKN was distinguished from other samples due to the highest contents of tZR and Z (Figure 1B). BN was characterized by the relatively high level of SA, while RN was characterized by the high level of ABA (Figure 1B). Hence, the biosynthesis of phytohormones in tea leaves was differentially regulated under different shading treatments.

### Effects of Shading Treatments on the Contents of Flavonoids in Tea Leaves

Table 2 shows the contents of catechins and flavonol glycosides in the tea leaves under different shading treatments. RN contained the highest content of total catechins (TC) at 138.6 mg/g dry weight (DW), subsequently followed by BN (135.7 mg/g DW) and BKN (133.0 mg/g DW), while CK contained the lowest content of TC at 128.4 mg/g DW. CK contained the highest level of total flavonol glycosides (TFG), followed by RN, while BN and BKN contained the lowest levels of TFG being around 3.28 mg/g. The shading treatment (95% shade percentage) slightly elevated the content of TC by 3.6–7.9% compared with CK, while it sharply reduced the content of TFG by 51.6–64.6%. The increase in TC was mainly attributed to the increase in EGCG, with the increased percentage being 15%. Thus, flavonol glycosides were more sensitive to the growth light condition than catechin compounds, which was consistent with the previous studies (Jin et al., 2021; Ye et al., 2021). The flavonoid composition of BN was close to that of BKN, suggesting that blue net shading treatment exerted a similar effect on flavonoid compounds in tea leaves to black net shading treatment. The comparable effects of blue and black net shading treatments on the reduction of flavonol glycosides in tea leaves have been reported (Jin et al., 2021).

**Table 2:** The contents of catechins and flavonol glycosides in the fresh tea leaves under different shading treatments.

**Compounds^**a**^**	**CK**	**BKN**	**BN**	**RN**
**Catechins (mg/g dry weight)**				
GC^b^	1.42 ± 0.08a	0.93 ± 0.05b	0.81 ± 0.04c	1.02 ± 0.05b
EGC^b^	17.58 ± 0.27c	16.91 ± 0.16d	18.47 ± 0.11b	24.16 ± 0.21a
C^b^	1.70 ± 0.08a	1.10 ± 0.09c	1.37 ± 0.15b	1.18 ± 0.19bc
EC^b^	9.22 ± 0.16a	6.55 ± 0.04c	7.03 ± 0.05b	2.32 ± 0.03d
EGCG^b^	75.47 ± 0.38b	87.13 ± 0.71a	88.31 ± 0.72a	87.78 ± 0.41a
GCG^b^	0.27 ± 0.01b	0.32 ± 0.01a	0.28 ± 0.02b	0.21 ± 0.01c
ECG^b^	22.31 ± 0.24a	19.86 ± 0.20c	19.28 ± 0.18d	20.93 ± 0.29b
CG^b^	0.42 ± 0.01b	0.19 ± 0.01c	0.14 ± 0.01d	0.97 ± 0.04a
TC^d^	128.39 ± 0.31d	132.99 ± 0.76c	135.69 ± 1.07b	138.56 ± 0.72a
	(100.0%)	(103.58%)	(105.68%)	(107.91%)
**Flavonol glycosides (μg/g dry weight)**				
M-gal-rha-glu^c^	200 ± 15a	146 ± 7b	146 ± 8b	158 ± 14b
M-gal^c^	2524 ± 37a	1007 ± 5d	1132 ± 41c	1450 ± 16b
M-glu^c^	1592 ± 5a	347 ± 1d	416 ± 12c	587 ± 8b
Q-gal-rha-glu^c^	1205 ± 7a	283 ± 2c	249 ± 1d	475 ± 5b
Q-glu-rha-glu^c^	1102 ± 6a	167 ± 3c	152 ± 1c	268 ± 14b
Q-glu-rha-rha^c^	564 ± 29b	621 ± 3a	504 ± 18c	572 ± 8b
Q-glu-rha^c^	228 ± 13a	45 ± 1c	50 ± 4c	76 ± 4b
Q-gal^c^	804 ± 5a	141 ± 2c	167 ± 3c	262 ± 7b
Q-glu^c^	258 ± 1a	48 ± 1c	54 ± 1c	80 ± 1b
K-glu-rha-glu^c^	533 ± 6a	335 ± 4c	281 ± 10d	380 ± 7b
K-gal^c^	125 ± 4a	94 ± 4c	75 ± 6c	108 ± 2b
K-glu-rha^c^	46 ± 2a	27 ± 2b	22 ± 3c	27 ± 1b
K-glu^c^	52 ± 4a	21 ± 0c	21 ± 1c	30 ± 1b
TFG^d^	9233 ± 91a	3281 ± 19c	3270 ± 20c	4473 ± 80b
	(100%)	(35.53%)	(35.42%)	(48.44%)

^a^*EC, (–)-epicatechin; EGC, (–)-epigallocatechin; ECG, (–)-epicatechin gallate; EGCG, (–)-epigallocatechin gallate; GC, (+)-gallocatechin; C, (+)-catechin; TC, total catechins; M-gal-rha-glu, myricetin galactosyl-rhamnosyl-glucoside; M-gal, myricetin galactoside; M-glu, myricetin glucoside; K-glu-rha-gal, kaempferol glucosyl-rhamnosyl-galactoside; K-gal, kaempferol galactoside; K-glu-rha, kaempferol glucosyl-rhamnoside; K-glu, kaempferol glucoside; Q-gal-rha-glu, quercetin galactosyl-rhamnosyl-glucoside; Q-glu-rha, quercetin glucosyl-rhamnoside; Q-glu-rha-rha, quercetin glucosyl-rhamnosyl-rhamnoside; Q-glu-rha-glu, quercetin glucosyl-rhamnosyl-glucoside; Q-gal, quercetin galactoside; Q-glu, quercetin glucoside; TFG, total flavonol glycosides; CK, tea sample grown in natural light; BKN, black net shade-treated sample; BN, blue net shade-treated sample; RN, red net shade-treated sample. Data with different alphabetic letters (a, b, c, d) in the same row were significantly different at p < 0.05. Significant difference analysis was carried out by the SAS System for Windows version 8.1 (SAS Institute Inc., Cary, NC, USA) using Tukey's test. Data were expressed as mean ± SD (n = 3)*.

^b^*Quantified by the corresponding authentic standards*.

^c^*Relatively quantified by the corresponding aglycone*.

^d^*Data in brackets are the percentage of TC or TFG compared with CK*.

### Transcriptome Profiles of Different Tea Samples and KEGG Enrichment Analysis

**Figure 2:**
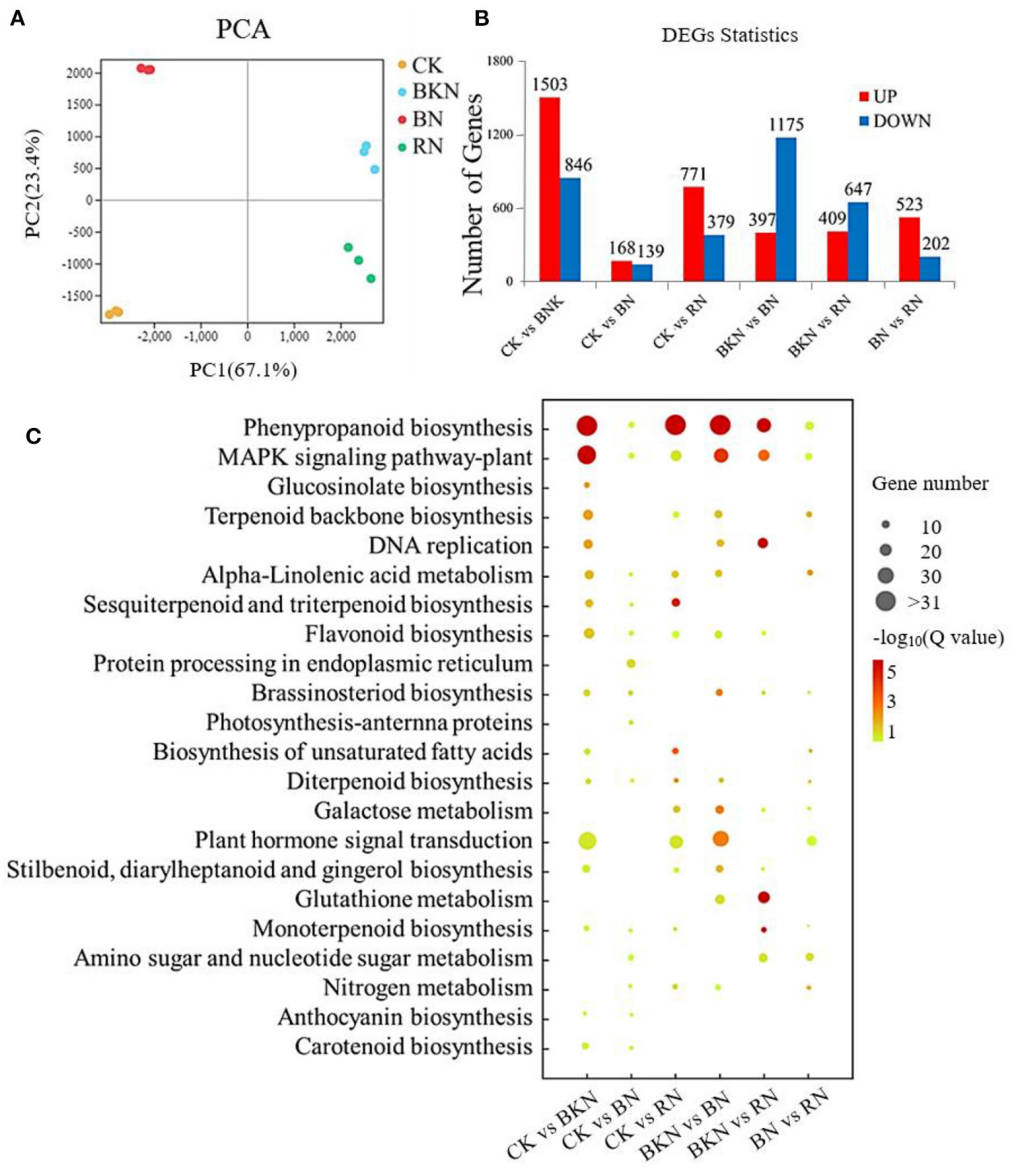
The gene expression profiles of the 2nd tea leaves. **(A)** PCA analysis; **(B)** DEG numbers; **(C)** the enriched KEGG pathways of DEGs. The number of replicates is 3.

Supplementary Table 3 shows the information of the RNA-Seq data. There were 45.0–51.4 million, 46.1–52.9 million, 36.5–48.0 million, and 43.1–49.9 million RNA-Seq clean paired-end reads, respectively, obtained for control, BKN, BN, and RN (Supplementary Table 3). Figure 2A is the PCA score plot of RNA-Seq data, and the samples under different shading treatments were clustered and well discriminated from each other. The new genes are listed in Supplementary Table 4. The results of gene expression obtained by RNA-Seq were validated by RT-qPCR, with *R*^2^ being 0.823 (Supplementary Figure 2). This suggests that the RT-qPCR results were highly correlated with the corresponding transcriptomic data, and the transcriptomic dataset was able to represent the transcript abundances. Figure 2B shows the number of upregulated and downregulated DEGs in different tea samples compared in pairs. The pair of CK vs. BN had the lowest number of DEGs (168 upregulated and 139 downregulated DEGs), while the pair of CK vs. BKN had the highest number of DEGs (1,503 upregulated and 846 downregulated DEGs), subsequently followed by the pair of BKN vs. BN (397 upregulated and 1,175 downregulated DEGs). This was consistent with the result in Figure 2A. The DEGs of different comparison pairs were analyzed by KEGG functional enrichment. In Figure 2C, phenylpropanoid biosynthesis and MAPK signaling pathways were significantly enriched in the pairs of CK vs. BKN, CK vs. RN, BKN vs. BN, and BKN vs. RN, while only protein processing in the endoplasmic reticulum and brassinosteroid biosynthesis pathways was enriched in the pairs of CK vs. BN. This suggests that the least number of enriched pathways was observed for the pairs of CK vs. BN. In particular, the pathway of plant hormone signal transduction was enriched in the pairs of BKN vs. BN, CK vs. BKN, and CK vs. RN, which was in an agreement with the PCA result that BN had the shortest distance from CK based on the composition of phytohormones (Figure 1A). Hence, different shading treatments could differentially regulate the hormone signal transduction in tea leaves.

### Phytohormone Biosynthesis Network

**Figure 3:**
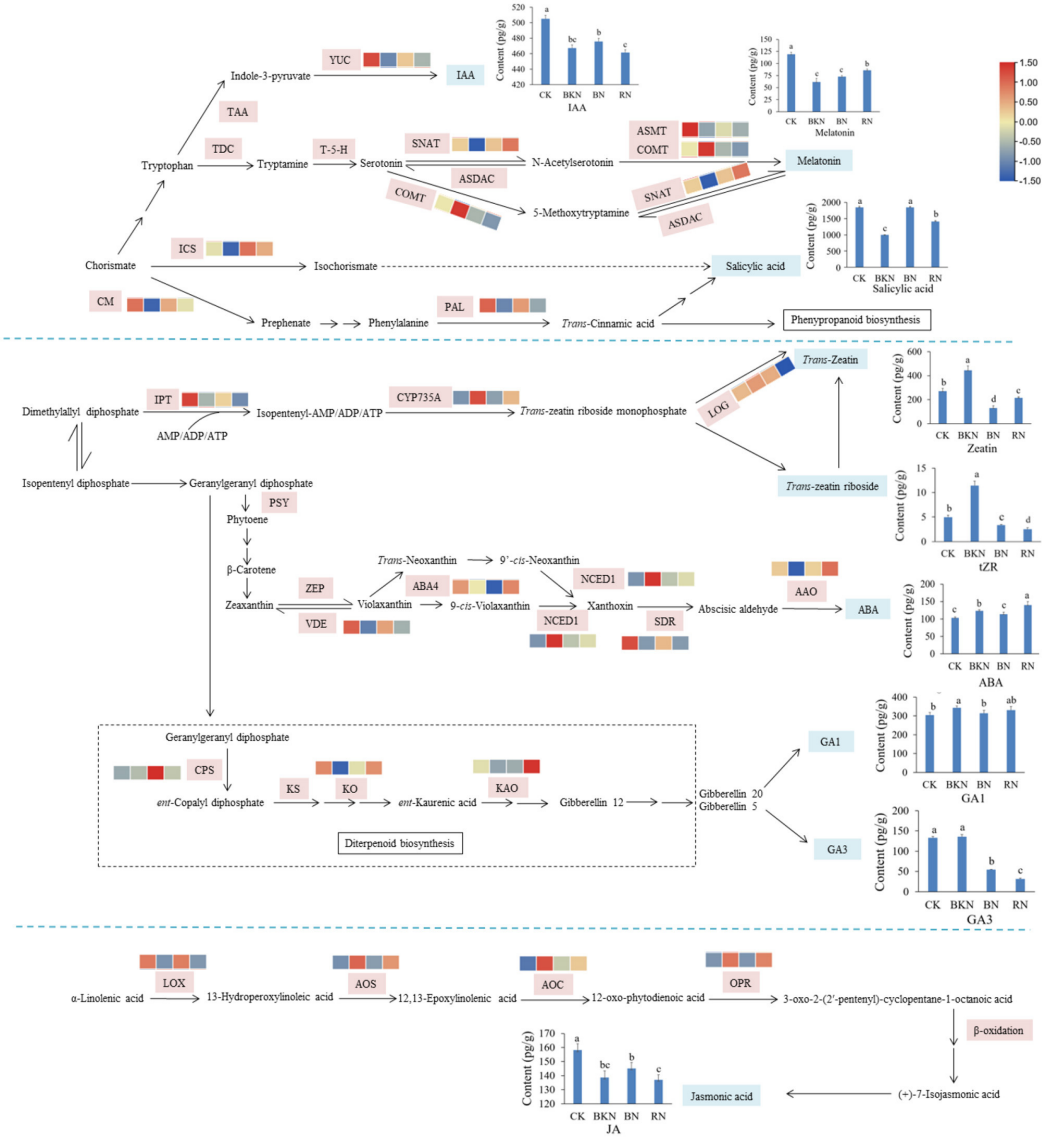
Visualization of the expression levels of the structural genes in the biosynthetic pathway of phytohormones in tea leaves. The contents of 8 detected phytohormones annotated with their structures are shown by line charts. CK, tea sample without shade; BKN, black net shade-treated sample; BN, blue net shade-treated sample; RN, red net shade-treated sample. PAL, phenylalanine ammonia-lyase; CM, chorismate mutase; ICS, isochorismate synthase;YUC:indole-3-pyruvate monooxygenase; IPT, adenylate isopentenyltransferase; CYP735A, cytokinin hydroxylase; CYP714B2, cytochrome P450 714B2; LOG, cytokinin riboside 5′-monophosphate phosphoribohydrolase; AMP, adenosine 5′-monophosphate; NCED, 9-*cis*-epoxycarotenoid dioxygenase; SDR, short chain dehydrogenase/reductase; MoCo, molybdenum cofactor; CYP707A, abscisic acid 8′-hydroxylase CYP707A; SNAT, serotonin-N-acetyltransferase; ASMT, N-acetylserotonin methyltransferase; COMT, caffeic acid-*O*-methyltransferase; CPS, *ent*-copalyl diphosphate synthase; KO, *ent*-kaurene oxidase; KAO, *ent*-kaurenoic acid oxidase; 20ox, Gibberellin 20; LOX, lipoxygenase; AOS, allene oxide synthase; AOC, allene oxide cyclase; OPR, 12-oxophytodienoate reductase; JAR, jasmonoyl-L-amino acid synthetase; JMT, jasmonate *O*-methyltransferase.

Figure 3 shows the biosynthetic pathways of the major phytohormones in tea leaves, with the annotation of transcriptional levels of structural genes. IAA, melatonin, and SA are originated from the same compound chorismate, and the key structural genes in the phytohormone biosynthetic pathways were affected by different light conditions. For example, the transcription levels of indole-3-pyruvate monooxygenase (YUC), the key enzyme for the biosynthesis of IAA, were downregulated by 0.61–0.83-fold in the shading-treated samples compared with CK. The expression of *YUC* in BN was higher than those of BKN and RN, which generally agreed with the levels of IAA in different samples. Great difference in gene expressions was also observed in *serotonin-N-acetyltransferase (SNAT), caffeic acid-O-methyltransferase (COMT)*, and *N-acetylserotonin methyltransferase (ASMT)* involved in the biosynthesis of melatonin. The lowest expression levels of *isochorismate synthase (ICS)* and *chorismate mutase (CM)* were observed in the BKN samples, which was consistent with the lowest content of SA in BKN. Only the expressions of *cytokinin hydroxylase (CYP735A)* showed a concordant change trend with the levels of Z and tRZ in different tea samples. No obvious correlation was observed in other biosynthetic genes of Z and tZR. A similar phenomenon was also observed in ABA, Gas, and JA that the levels of these three phytohormones were not apparently correlated with the transcription levels of key structural genes.

### Association of Phytohormone Biosynthesis With Light Signal Transduction

**Figure 4:**
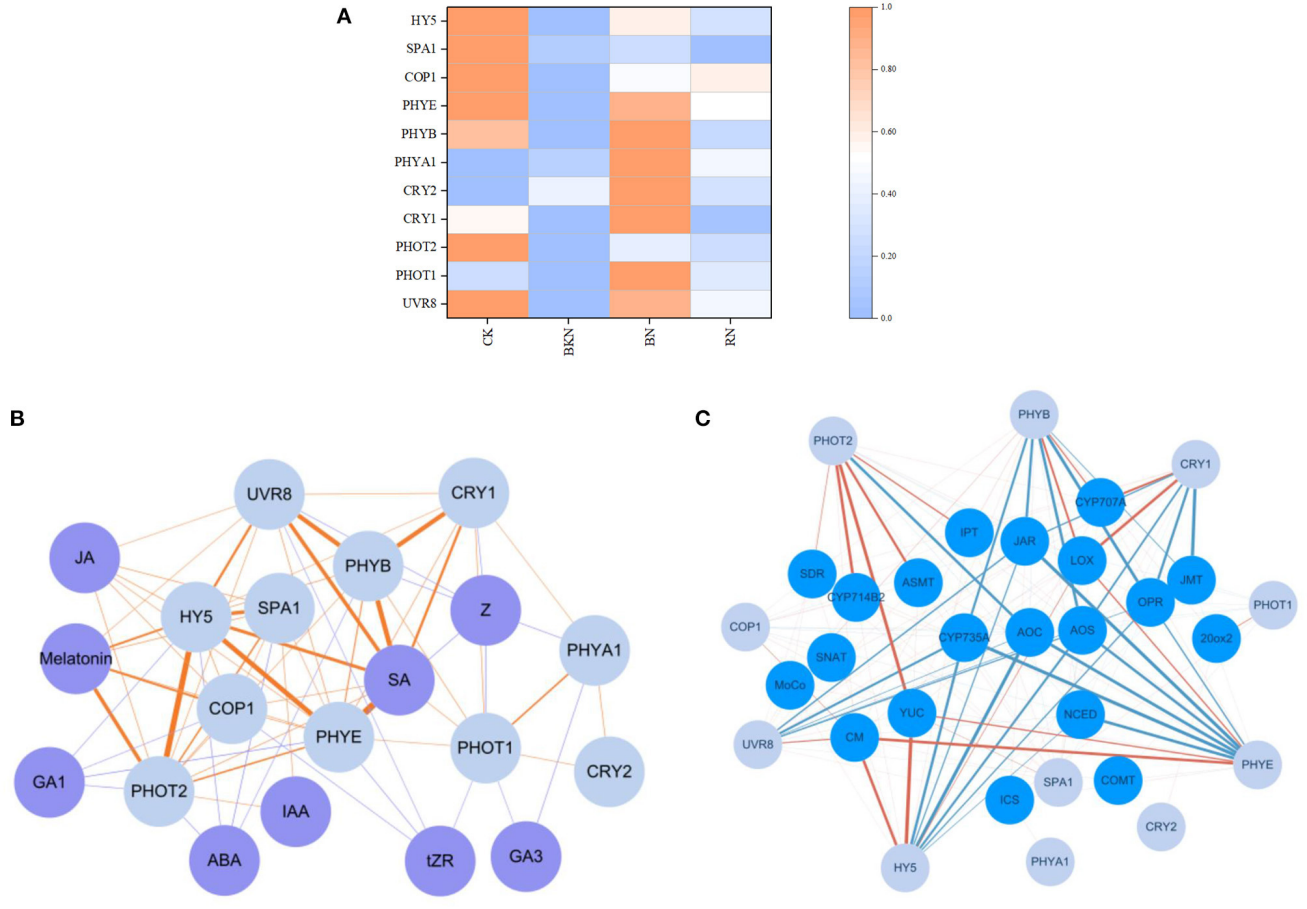
The effects of different shading treatments on the expressions of light signal and phytohormone biosynthesis-related genes in tea leaves. **(A)** The heatmap of light signal-related genes, **(B)** the correlation network of light signal-related genes (light blue nodes) and phytohormone levels (purple nodes), and **(C)** the correlation network of light signal-related genes (light blue nodes) and phytohormone biosynthetic genes (blue nodes). The co-expression networks of the FPKM values of light signal perception and signaling genes (PHYB, PHYE, PHOT1, CRY1, CRY2, UVR8, COP1, SPA1, and HY5) and the phytohormone contents (ABA, GA1, GA3, IAA, JA, melatonin, SA, tZR, and Z), as well as the FPKM values of light signal perception and signaling genes (PHYB, PHYE, PHOT1, CRY1, CRY2, UVR8, COP1, SPA1, and HY5) and the phytohormone biosynthetic genes (CM, ICS, YUC, IPT, CYP735A, CYP714B2, NCED, SDR, MoCo, CYP707A, SNAT, ASMT, COMT, 20ox2, LOX, AOS, AOC, OPR, JAR, and JMT), were conducted using the Cytoscape software (version 3.8.0). Significant correlation was presented based on the statistical test with a robust cutoff (*p* < 0.05). The orange color lines represented positive correlation and the blue color lines represented negative correlation. The correlation coefficient increased from 0.80 to 0.99 as the line color deepened. HY5, Elongated hypocotyl 5; SPA1, suppressor of PHYA; COP1:constitutive photomorphogenesis 1; PHYs, phytochromes; CRYs, cryptochromes; UVR8, UV resistance locus 8; ABA, abscisic acid; GA1, gibberellin A1; GA3, gibberellin A3; IAA, indole-3-acetic acid; JA, jasmonic acid; SA, salicylic acid; tZR, *trans*-zeatin-riboside; Z, zeatin; CM, chorismate mutase; ICS, isochorismate synthase; YUC, indole-3-pyruvate monooxygenase; IPT, adenylate isopentenyltransferase; CYP735A, cytokinin hydroxylase; CYP714B2, cytochrome P450 714B2; NCED, 9-*cis*-epoxycarotenoid dioxygenase; SDR, short chain dehydrogenase/reductase; MoCo, molybdenum cofactor; CYP707A, abscisic acid 8′-hydroxylase CYP707A; SNAT, serotonin-N-acetyltransferase; ASMT, N-acetylserotonin methyltransferase; COMT, caffeic acid-*O*-methyltransferase; 20ox2, gibberellin 20; LOX, lipoxygenase; AOS, allene oxide synthase; AOC, allene oxide cyclase; OPR, 12-oxophytodienoate reductase; JAR, jasmonoyl-L-amino acid synthetase; JMT; jasmonate *O*-methyltransferase; CK, tea sample grown in nature light; BKN, black net shade-treated sample; BN, blue net shade-treated sample; RN, red net shade-treated sample. Each cell represents the mean value of 3 replicates.

Figure 4A shows the heatmap of light signal-related gene expressions under different shading treatments. Obviously, CK generally had the highest expressions of light receptors and signal transduction genes, subsequently followed by BN and RN, while BKN had the lowest expressions. This was in a general agreement with the radiation intensities under different shading treatments. Specifically, *CRY1, CRY2*, and *PHOT1* that perceive blue light were transcriptionally upregulated under blue net shading treatment, compared with CK. However, the transcriptional levels of light signal transduction-related genes *HY5, SPA1*, and *COP1* in BN were much lower than those in CK, despite the relatively high expressions of light receptors. Thus, the higher expressions of light receptor genes in CK than BN, such as *UVR 8* and *PHYE*, might be more related to the light signal transduction of *HY5, SPA1*, and *COP1*. The correlation network of light signal-related genes and phytohormone levels is shown in Figure 4B. Among these nine endogenous phytohormones, the contents of SA and melatonin were highly positively correlated with the expressions of light receptor and signal transduction genes, with Pearson's coefficients being above 0.80. The transcriptional levels of several biosynthetic genes also showed correlations with the expressions of light receptor and signal transduction genes (Figure 4C). *UVR8, PHOT2, PHYB, CRY1, PHYE*, and *HY5* had more close interactions than other light signal-related genes. This is generally consistent with the direct or indirect correlations in Figure 4B. *CM, YUC, cytochrome P450 714B2 (CYP714B2), ASMT, lipoxygenase (LOX)*, and *CYP707A* were highly positively correlated with the expressions of the corresponding light signal-related genes, while *CYP735A*, allene oxide cyclase (*AOC*), *allene oxide synthase (AOS), JAR, OPR, NCED*, and *JMT* had highly negative correlations (Figure 4C).

## Discussion

The intensity and quality of light are important factors in the biomass and metabolism of plants (Fan et al., 2013; Chang and Chang, 2014; Kaiser et al., 2019). The growth of plants is accelerated, and the biomass of crops is increased under the appropriate light condition (Fu et al., 2017). In field management, prolonging illumination duration and supplementary lighting are propitious to the production yield of plants (Bian et al., 2016; Zheng et al., 2018). Our study showed that the biomass of young shoots of “Longjing 43” was affected by different shading treatments, which was positively correlated with radiation intensity. Despite the great difference in the biomass between the black net-treated sample and the colored net-treated samples, the contents of flavonol glycosides were greatly reduced under all the shading treatments. This was consistent with our previous studies (Jin et al., 2021; Ye et al., 2021). The transcriptions of light signal-related genes were also regulated by different shading treatments. In this study (tea plants “Longjing 43”), the expressions of light signal-related genes in BN were relatively higher than those in BKN and RN, including *HY5, PHYE, PHYB, PHYA1, CYR1, CYR2, PHOT1, PHOT2*, and *UVR8*. Higher expressions of *HY5, PHYE, PHYB*, and *CRY1* were also reported in tea plants “Fudingdabaicha” under blue net-shading treatment, compared with black net and red net-shading treatments (Ye et al., 2021). The slight difference in upregulated transcriptions of light signal-related genes could be attributed to the cultivar difference. Menendez et al. (2021) associated the expression of *HY5* with the production yield of crops. Our study showed that CK had the highest production yield of young shoots, subsequently followed by BN and RN, while BKN had the lowest yield. The higher production yields of BN and RN were mainly due to the greatly increased bud density and elevated bud weight under blue and red net shading treatments, compared with BKN. Since the shading treatments were carried out shortly after light pruning, the growth stage of tea plants was normalized among different treatments. The great increase in bud density and bud weight could be attributed to the phytohormone changes under different light conditions. According to the result of phytohormone analysis, different samples had different phytohormone profiles. The accumulation of SA and ABA might be more sensitive to light quality, compared with other phytohormones. The impact of light quality on the biomass production of rice has also been reported, which was associated with the change in net assimilation rate (Ohashi-Kaneko et al., 2006).

The biosynthesis of phytohormones in plants is regulated by light condition (Yang and Li, 2017; Yadav et al., 2020). Our study investigated the correlations between phytohormone levels and the expressions of light signal-related genes and found that SA was closely correlated with the expression levels of *UVR8, PHYB, HY5, PHYE*, and *CRY1*, while melatonin had correlations with *PHOT2, HY5*, and *COP1*. Kurepin et al. (2010) reported that the level of SA was regulated by light in the hypocotyls of *Helianthus annuus* seedlings. SA is also involved in the radiation-induced stress of plants (Uppalapati et al., 2007). PHYB was reported playing an important role in the SA-induced defenses of phyb mutant (de Wit et al., 2013). Besides, UV-B not only regulated the SA-induced signal transduction (Demkura et al., 2010) but also promoted the accumulation of melatonin in plants (Hardeland, 2016). Our result also showed that the levels of SA and melatonin in the 2nd tea leaves were more affected by the intensities of blue light and UV, compared with other phytohormones. However, no great correlation was observed between the expressions of light signal-related genes and the levels of other phytohormones, which might be associated with the leaf samples collected. Since the 2nd leaves were collected for transcriptome analysis, and the biosynthesis of IAA, GAs, Z, and tRZ may be largely attenuated in the expanding leaves. The biosynthesis of IAA, GAs, Z, and tZR is especially vigorous in the meristematic tissues, such as the growing tips or apical of stems and roots, while their biosyntheses are attenuated in the mature and developing organs. Besides, the transcriptional levels of relevant structural genes showed no obvious coordination with the levels of ABA, GAs, and JA, since the transportation and transformation of phytohormones also importantly regulate the phytohormone levels in plants in addition to biosynthesis. The effects of light condition on the transportation and transformation of phytohormones need attention in the future study.

## Data Availability Statement

The datasets presented in this study can be found in online repositories. The names of the repository/repositories and accession number(s) can be found below: https://bigd.big.ac.cn/, No. PRJCA008920.

## Author Contributions

JJ and J-HY designed the research. Z-TF wrote the manuscript. W-ZH, Z-FS, J-NS, and JJ conducted the field experiment. Z-FS, J-NS, and YY collected the experimental materials and did the analysis. J-LL and Z-SF did the supervision. J-HY and J-LL revised the manuscript. All authors listed here contributed and approved the manuscript.

## Funding

This study was financially supported by the Zhejiang Science and Technology Major Program on Agricultural New Variety Breeding-Tea Plant (2021C02067-5), the Major Agricultural Technology Collaborative Extension Project of Zhejiang Province (2020XTTGCY04), and the Zhejiang Province Six Aspects of Agriculture, Rural areas and Farmers Science and Technology Cooperation Project (2021SNLF014).

## Conflict of Interest

Z-SF is employed by the Zhejiang Minghuang Natural Products Development Co., Ltd. The remaining authors declare that the research was conducted in the absence of any commercial or financial relationships that could be construed as a potential conflict of interest.

## Publisher's Note

All claims expressed in this article are solely those of the authors and do not necessarily represent those of their affiliated organizations, or those of the publisher, the editors and the reviewers. Any product that may be evaluated in this article, or claim that may be made by its manufacturer, is not guaranteed or endorsed by the publisher.
