# Supplementary material for: Effects of Different Shading Treatments on the Biomass and Transcriptome Profiles of Tea Leaves (Camellia sinensis L.) and the Regulatory Effect on Phytohormone Biosynthesis
Source: Front Plant Sci. 2022 Jun 24;13:909765. doi: 10.3389/fpls.2022.909765 (PMC9266624; doi:10.3389/fpls.2022.909765)
Supplement: Supplementary file 1 [file Data_Sheet_1.docx]

**Table S1**| The primers sequence for qPCR and promoter cloning

| **Gene** | **Primer (5’**$\boldsymbol{\to}$**3’)** | |
| --- | --- | --- |
| β-actin | F | CTTCCTCATGCTATCCTCCGTCTT |
|  | R | ATTTCCCGTTCAGCAGTGGTG |
| PAL | F | ACGACAACCCCTTGATCGAC |
|  | R | TTGATGCCAAAGCCAGCCTA |
| 4CL | F | TTCATGCGGAACTGTGGTCA |
|  | R | TGGAGCCAACCATCCACATC |
| CHS | F | GCAGGACATGGTTGTGGTTG |
|  | R | TTGACTGATGGGCGAAGACC |
| MYB86 | F | GGTCTCCTGAAGAAGATGAGAAG |
|  | R | CTTTGCAAGCCTGCTAGTTTAG |
| F3H | F | ACTCAAGATGGCCCGACAAG |
|  | R | CCTTCTCAAGGCCCATAGCC |
| HY5 | F | GAGTTGGAGGGTAGGGTTAAAG |
|  | R | CATTCCTGCTGTTGTGTTCTTC |
| MYB12 | F | GATTGTAGGCAGTCCTCATCAC |
|  | R | CAACATCCCACTCTCATCTAACC |
| ANS | F | AACAAGCGAGTACGCAAAGC |
|  | R | TGAAGCTCTTCCATGCCTCC |
| FLS | F | CCCTCGGAGTTGAACCTCAC |
|  | R | ACGACAAACACAGCCCAAGA |

**TABLE S2** |The contents of phytohormones in tea plants under different shading treatments (pg/g)

| Phytohormones | CK | BKN | BN | RN |
| --- | --- | --- | --- | --- |
| Abscisic acid | 102.91±2.84d | 123.17±3.16b | 113.62±5.49c | 140.02±9.98a |
| Gibberellin A1 | 305.00±13.13b | 343.90±8.71a | 314.54±14.98b | 330.35±19.78ab |
| Gibberellin A3 | 132.94±3.61a | 136.01±5.27a | 54.19±1.27b | 31.49±2.63c |
| IAA | 504.99±4.64a | 480.69±19.44bc | 475.75±4.04b | 461.36±3.83c |
| Jasmonic acid | 158.12±4.63a | 138.62±4.83bc | 145.19±4.18b | 137.10±3.41c |
| Melatonin | 119.10±4.27a | 61.71±7.61d | 72.93±2.28c | 86.05±2.37b |
| Salicylic acid | 1847.79±41.80a | 994.67±13.62c | 1851.59±29.66a | 1415.05±29.21b |
| *Trans*-zeatin-riboside | 4.98±0.38b | 11.41±0.95a | 3.34±0.16c | 2.55±0.32d |
| Zeatin | 270.81±21.96b | 444.36±38.10a | 130.20±19.55d | 216.03±8.95c |

^a^ *CK: control samples without shading treatment; BKN: black net shade-treated sample; BN: Blue net shade-treated sample; RN: red net shade-treated sample. Data were present as mean± SD (n = 3). Data with different alphabetic letters (a, b, c, d) in the column were significantly different at p<0.05.*

**Table S3 |** Statistics on the RNA-Seq data.

| Sample | Raw Data (bp) | Clean Data (bp) | Clean reads | Genes matched to reference genome |
| --- | --- | --- | --- | --- |
| CK-1 | 7731192300 | 7678120591 | 51414358 | 46707849 (91.22%) |
| CK-2 | 6773044500 | 6726139141 | 45030604 | 40879833 (91.10%) |
| CK-3 | 7702161600 | 7645777778 | 51211144 | 46497959 (91.05%) |
| BKN-1 | 7969030500 | 7915516201 | 52983484 | 48088985 (90.97%) |
| BKN-2 | 7586940000 | 7531639300 | 50436540 | 45715326 (90.85%) |
| BKN-3 | 6939653700 | 6888719549 | 46143866 | 41949208 (91.22%) |
| BN-1 | 5505371700 | 5456637193 | 36592868 | 33207389 (90.96%) |
| BN-2 | 7226399100 | 7168422516 | 48038844 | 43799679 (91.34%) |
| BN-3 | 7800377400 | 7737736943 | 51859422 | 47296002 (91.45%) |
| RN-1 | 7394444400 | 7342605291 | 49160140 | 44685563 (91.08%) |
| RN-2 | 6476133900 | 6420239419 | 43055394 | 39093960 (91.05%) |
| RN-3 | 7510437600 | 7456489910 | 49945968 | 45544888 (91.39%) |

**Figure S1**|Visible spectrum, light intensity, and UV intensity of different shading-treatment.


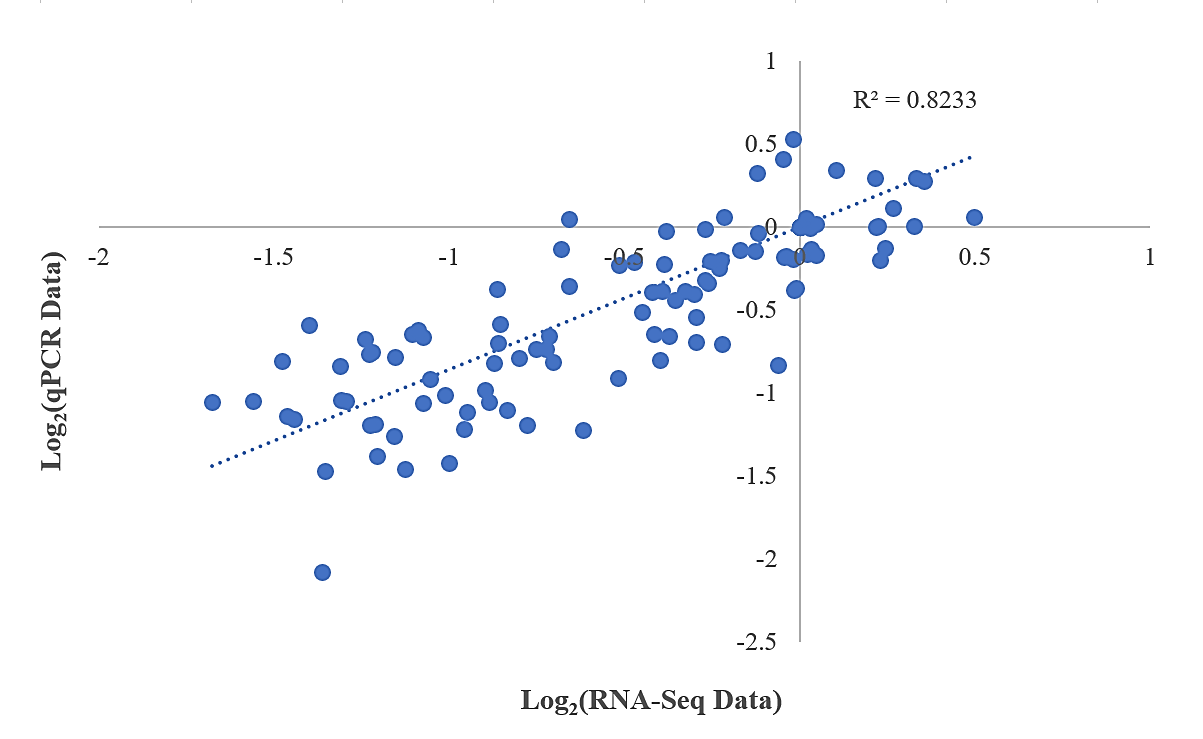


Log_2_ (qPCR Data)

Log_2_ (RNA-Seq Data)

**Figure S2**|The correlation between RNA-Seq and qPCR data
